# Supplementary material for: MRAP2 modifies the signaling and oligomerization state of the melanocortin-4 receptor
Source: Nat Commun. 2025 Sep 25;16:8324. doi: 10.1038/s41467-025-63988-w (PMC12462500; doi:10.1038/s41467-025-63988-w)
Supplement: Supplementary file 1 — Supplementary Information [file 41467_2025_63988_MOESM1_ESM.pdf]

SUPPLEMENTARY MATERIALS FOR

MRAP2 modifies the signaling and oligomerization state of the melanocortin-4 receptor

Iqra Sohail*, Suli-Anne Laurin*, Gunnar Kleinau, Vidicha Chunilal, Andrew Morton, Alfonso Brenlla, Zeynep Cansu Uretmen Kagiali, Marie-José Blouin, Javier A. Tello, Annette G. Beck-Sickinger, Martin J. Lohse, Patrick Scheerer, Michel Bouvier[#], Peter McCormick[#], Paolo Annibale[#], and Heike Biebermann[#]

This document contains:

Supplementary Table 1

Supplementary Figures 1-14

Supplementary Figures and Tables

Protein	nTMP	MC4R:MRAP2
MC4R	3.6	1:3
MRAP2	11.5	

Supplementary Table 1: The normalized gene expression values (nTMP) representing the RNA expression of the MC4R and MRAP2 in the paraventricular nucleus of the hypothalamus (The Human Protein Atlas, 2023). The values obtained give an expression ratio for MC4R to MRAP2.

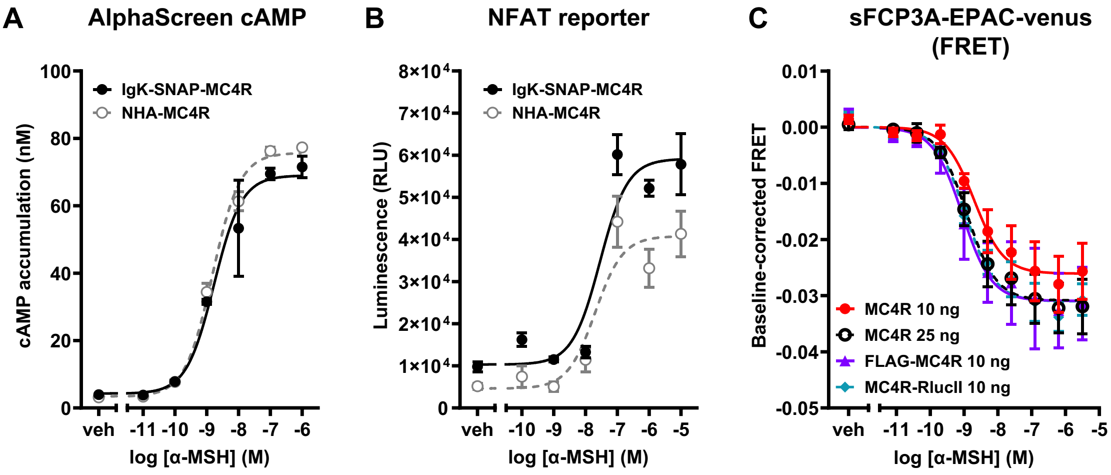

Supplementary Figure 1 : N-terminal and C-terminal tags used in this study do not impair MC4R downstream signaling. (A) N-terminal SNAP-tag doesn't impair cAMP accumulation, as assessed with AlphaScreen technology. **(B)** nor does it impair PLC activation, as assessed with NFAT-induced luminescence, as compared to N-terminal HA-tag MC4R (see Materials and Methods). **(C)** N-terminal FLAG-tag or C-terminal RlucII- tag do not impair cAMP production elicited by stimulation by α-MSH for 25 minutes compared to untagged MC4R, as assessed with sCFP3A-EPAC-Venus cAMP FRET biosensor. Data are expressed as mean ± SEM of three independent experiments.

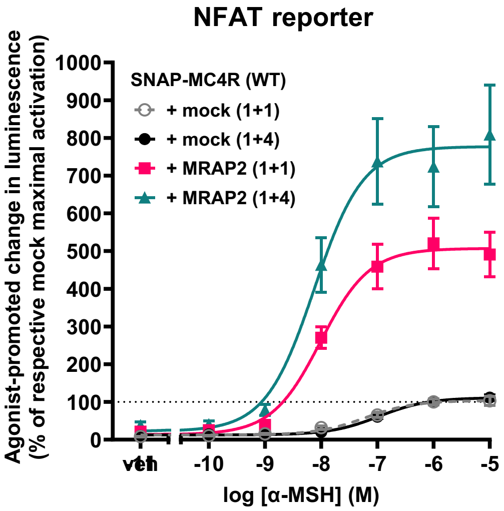

Supplementary Figure 2: Activation of phospholipase C activation via NFAT reporter gene assay. In order to determine the effect of MRAP2 on phospholipase C activation MC4R and indicated ratios of MRAP2 and NFAT reporter were co-transfected into HEK293 cells. 48 h after transfection cells were stimulated with increasing amounts of α-MSH for 6h and luciferase activity was determined. The result of 8 independent experiments performed in triplicates is shown (mean ± SEM).

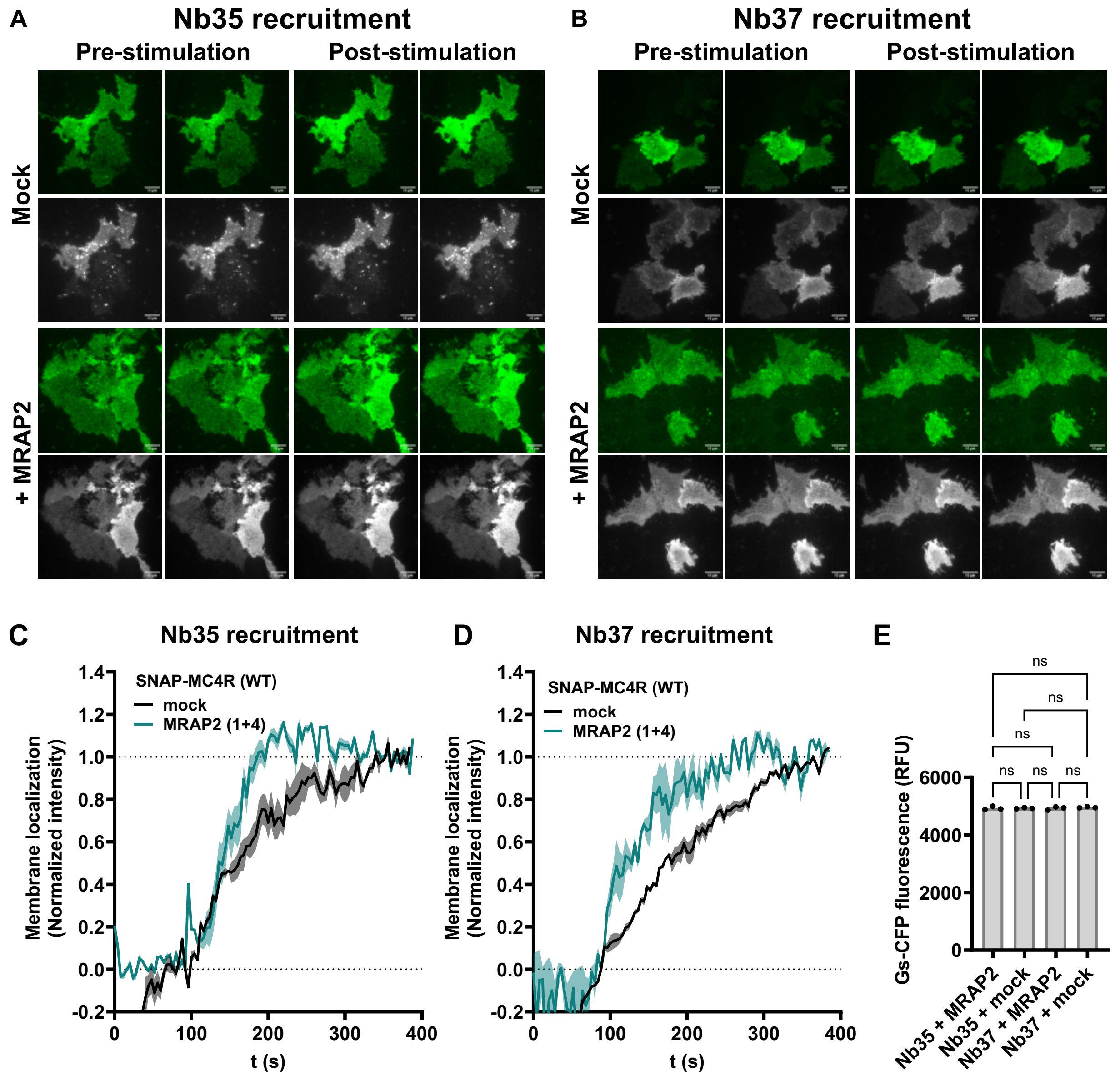

Supplementary Figure 3: Recruitment of Nb35 and Nb37 in HEK293 cells heterologously expressing SNAP-MC4R +/- MRAP2 and Gs. Recruitment of **(A)** Nb35-eYFP or **(B)** Nb37-eYFP to cerulean-Gs-positive cells with and without MRAP2 (1+4). SNAP-MC4R labeled with SNAP-Surface 647 (gray) and Nb35-eYFP (green). Kinetic of the intensity of **(C)** Nb35-eYFP or **(D)** Nb37-eYFP measured at the plasma membrane of HEK293AD cells using TIRF, with and without MRAP2. Shown are the normalized mean fluorescence intensities recorded at the membrane in the eYFP channel; the shaded area represents ±SEM from 3 individual experiments. **(E)** Fluorescence intensity (arbitrary intensity units) in the cyan channel from populations of cells co-transfected with Gs-CFP plasmid and the constructs indicated. Results are represented in bar chart as mean ± SEM, in which individual data point from the three individual experiments are shown as overlaid dot plot. Statistical analysis was performed using ordinary one-way ANOVA with Sidak's multiple comparisons post-hoc test. No fluctuation in the expression level of $G\alpha_s$ is observed (n=3).

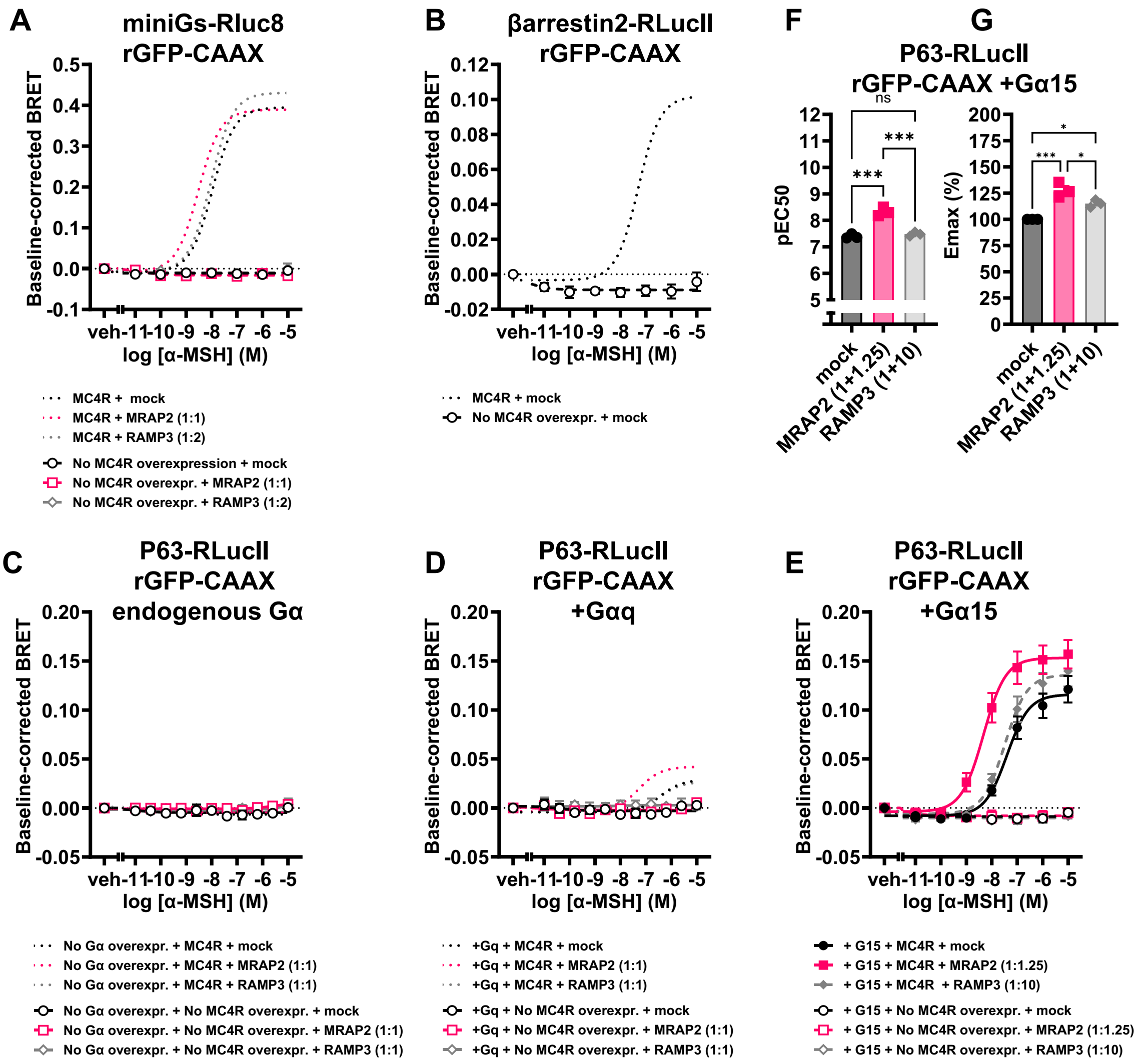

Supplementary Figure 4: Complementary BRET experiments. Mean concentration-response curves of BRET sensor stimulation with α-MSH, without or with overexpression of SNAP-MC4R in the same set of experiments unless stated otherwise, in the presence or absence of MRAP2 or RAMP3 at the indicated ratio with respect to MC4R, in transiently transfected HEK293-SL cells. Baseline-corrected (agonist-promoted change in BRET) responses are expressed as mean ± SEM of three independent experiments. **(A)** Rluc8-miniGs recruitment to the plasma membrane localization sensor rGFP-CAAX after 45 minutes of stimulation. Curves from **Figure 2C** are represented in dotted lines for scale reference. **(B)** β-arrestin2-RlucII recruitment to the plasma membrane localization sensor rGFP-CAAX after 3 minutes of stimulation. This experiment was carried independently of the one presented in the main text, with 300 ng of receptor. **(C-E)** Gq-family selective effector p63RhoGEF-RlucII recruitment to the plasma membrane localization sensor rGFP-CAAX after 45 minutes of stimulation, **(C)** in the presence or absence of overexpression of **(D)** $G\alpha_q$ or **(E)** $G\alpha_{15}$. Curves from **Figure 4A** are represented dotted lines for scale reference in panel D. **(F)** EC_{50} and **(G)** E_{max} from the p63RhoGEF recruitment experiments for G15. Results are represented in bar chart as mean ± SEM, in which individual EC_{50} and E_{max} data point from the three individual experiments are shown as overlaid dot plot. Statistical analysis was performed using ordinary one-way ANOVA with Tukey's multiple comparisons post-hoc test (* = $p < 0.05$ in which E_{max} MRAP2 vs RAMP3 adjusted *p*-value = 0.0170; E_{max} mock vs RAMP3 adjusted *p*-value = 0.0358,*** = $p < 0.001$ in which pEC_{50} mock vs MRAP2 adjusted *p*-value = 0.0002; pEC_{50} MRAP2 vs RAMP3 adjusted *p*-value = 0.0003; and E_{max} mock vs MRAP2 adjusted *p*-value = 0.0008).

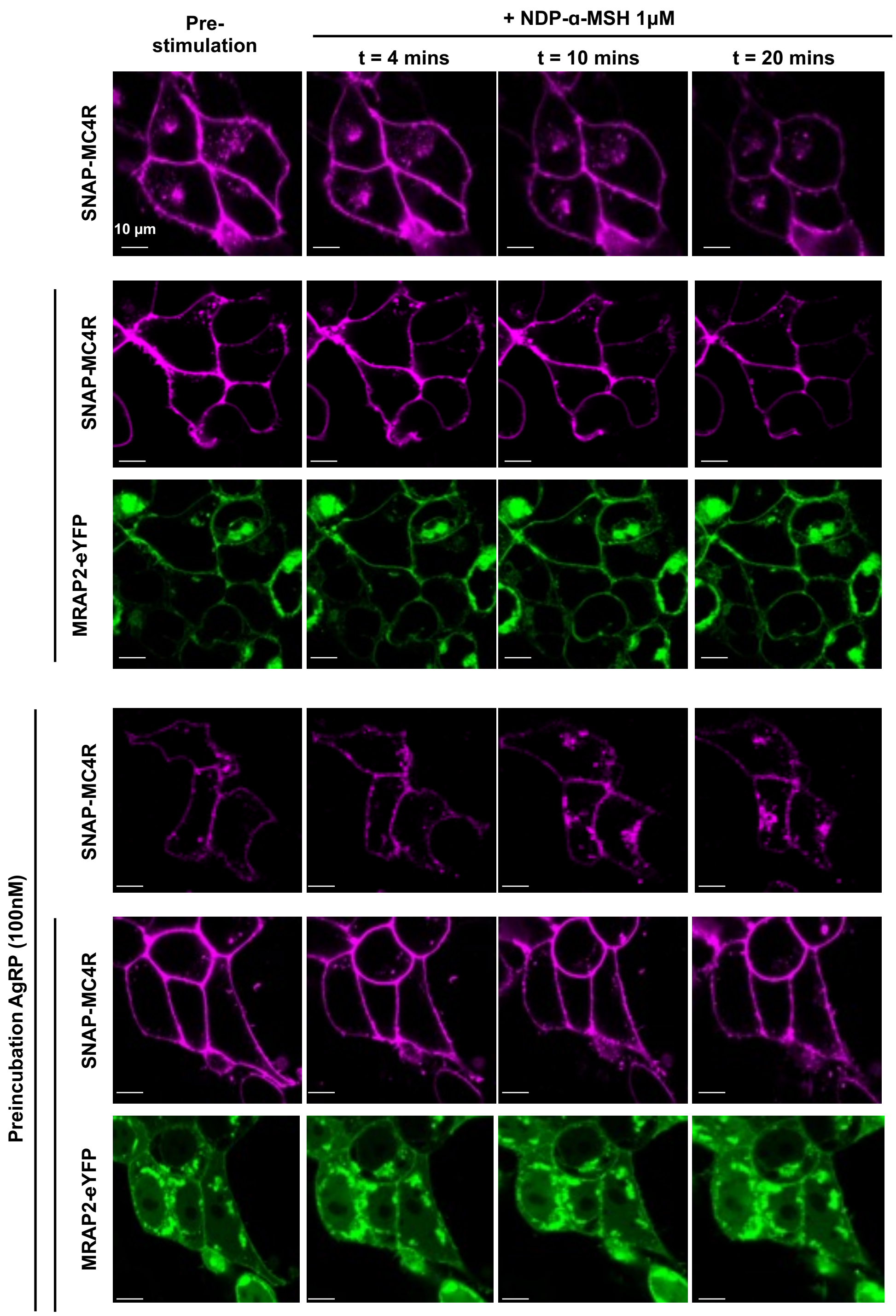

Supplementary Figure 5: Internalization of SNAP-MC4R with and without expression of MRAP2. From top to bottom, confocal micrographs of SNAP-MC4R together with eYFP-MRAP2, when indicated. Evolution of MC4R and MRAP2 cellular localization at four timepoints, before and respectively after 4, 10 and 20 minutes after the addition of 1μM NDP-α-MSH. In the bottom panel, cells expressing the receptor are further preincubated with 100 nM AgRP to suppress constitutive internalization. Scale bars are 10 μm.

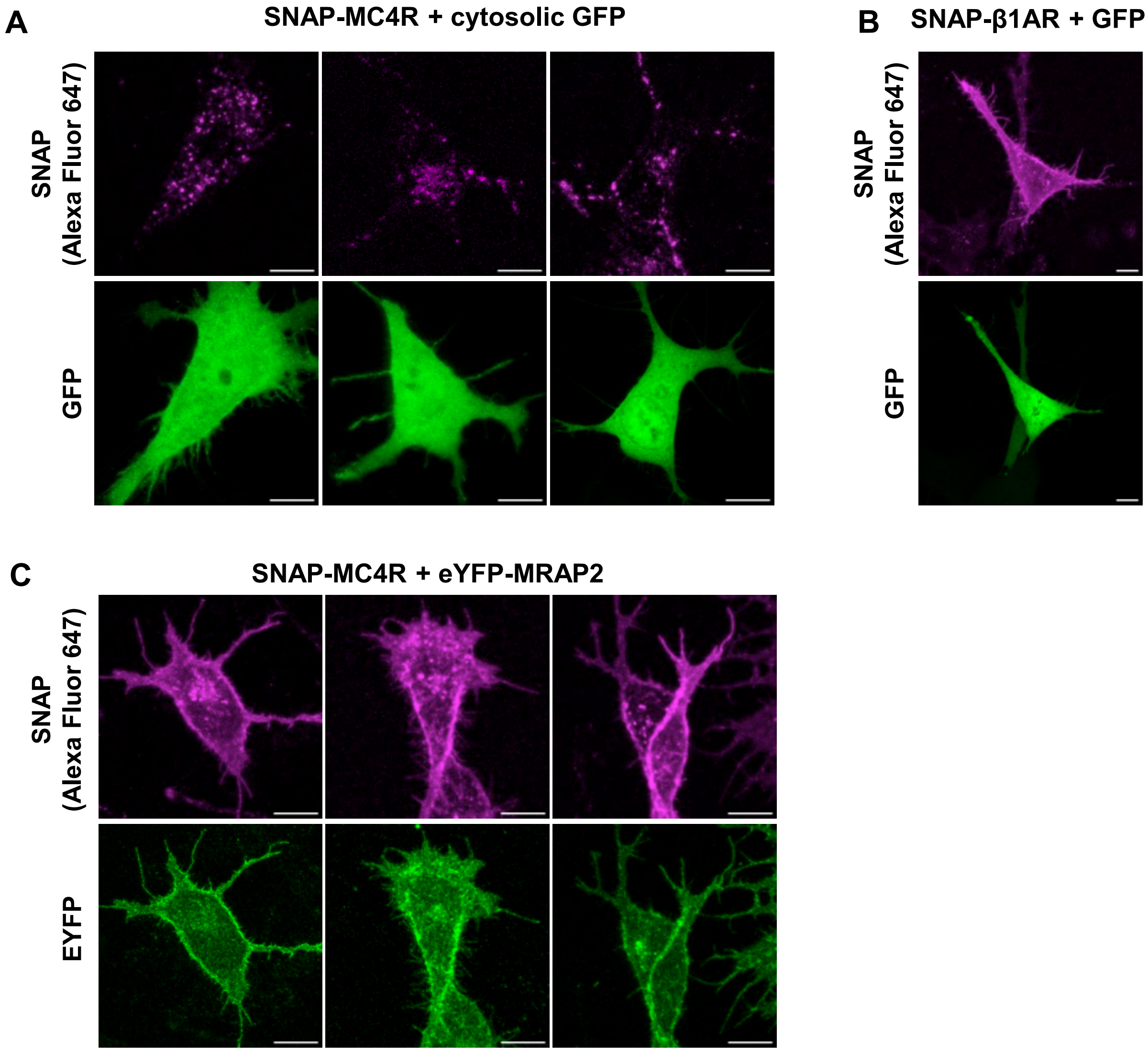

Supplementary Figure 6: Trafficking of SNAP-MC4R in the absence and presence of MRAP2 co-expression in N7 Embryonic Mouse Hypothalamus Cell. (A) Representative N7 cells electroporated with SNAP-MC4R and cytosolic GFP and further labeled with SNAP-Surface Alexa 647. **(B)** Representative N7 cells electroporated with SNAP-β1AR and cytosolic GFP and further labeled with SNAP-Surface Alexa 647. **(C)** Representative N7 cells electroporated with SNAP-MC4R and eYFP-MRAP2 and further labeled with SNAP-Surface Alexa 647. Confocal micrographs, scale bars are 10 μm.

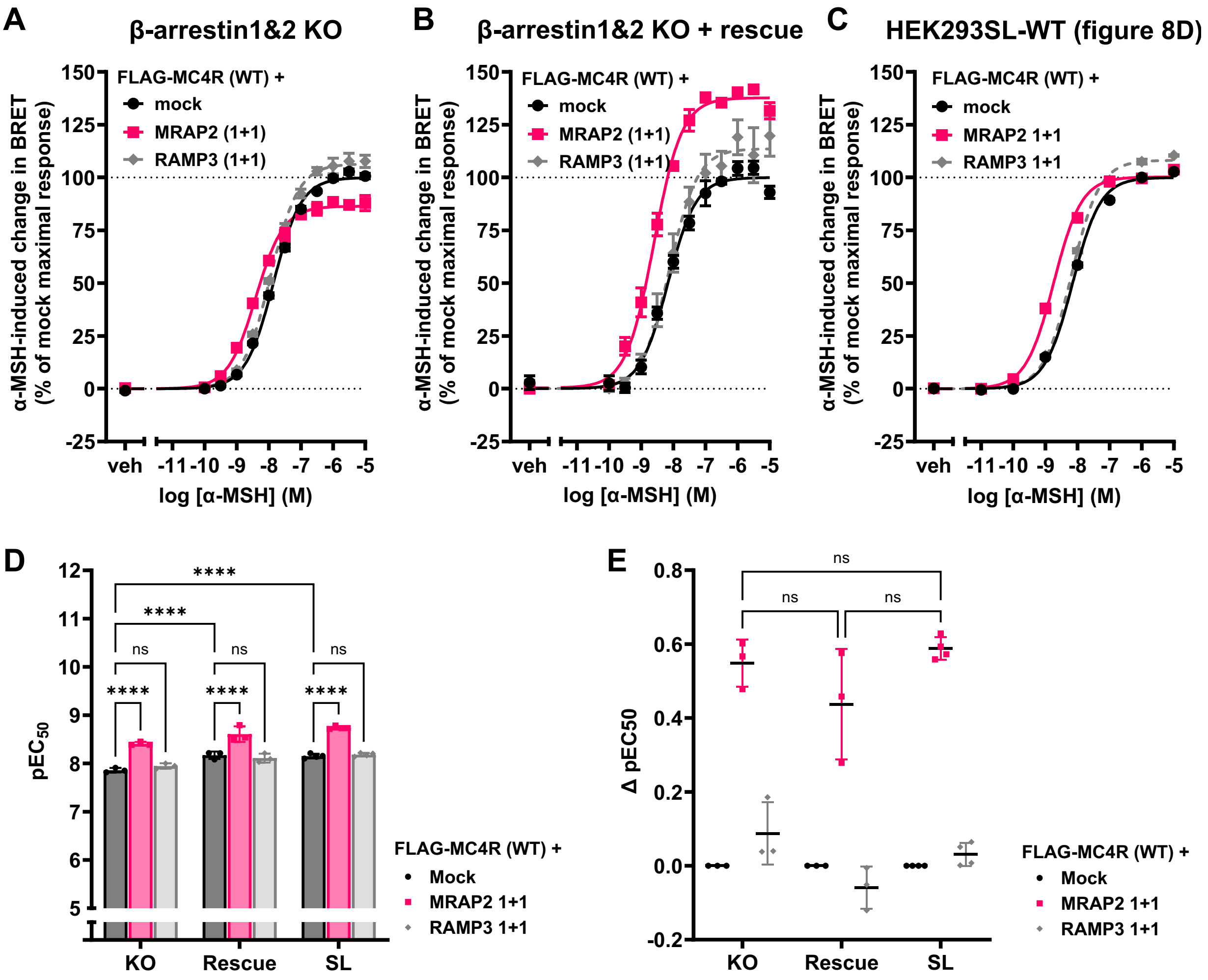

Supplementary Figure 7: MiniGs recruitment in β-arrestin1 and β-arrestin2 double knock-out cell. (A) Average concentration-response curves of Rluc8-miniGs recruitment to the plasma membrane localization sensor rGFP-CAAX upon MC4R stimulation with α-MSH for 45 minutes, in the presence or absence of MRAP2 or RAMP3, in transiently transfected β-arrestin1 and β-arrestin2 double knock-out cells. **(B)** β-arrestin1 and β-arrestin2 expression rescued the phenotype observed in related SL cells (not directly parental cells, as KO cells were maintained in FBS medium, while SL were maintained in NCS medium). **(C)** Data in HEK293SL from Figure 2C are shown again for easier comparison. Normalized data are expressed as mean ± SEM of three independent experiments. **(D)** pEC_{50} values from the miniGs recruitment BRET experiments in double KO cells, in absence (KO) or presence of the reintroduction of β-arrestins (rescue). Results are represented as mean ± SEM, in which individual pEC_{50} data point from the three individual experiments are shown.. pEC_{50} in HEK293-SL cells from **Figure 8D** are reported for comparison (SL). **(E)** Difference in pEC50 as compared to mock condition are displayed. Statistical analysis was performed using two-way ANOVA with Tukey's multiple comparisons post-hoc test (**** = $p < 0.0001$). Not all statistical results are shown for better clarity.

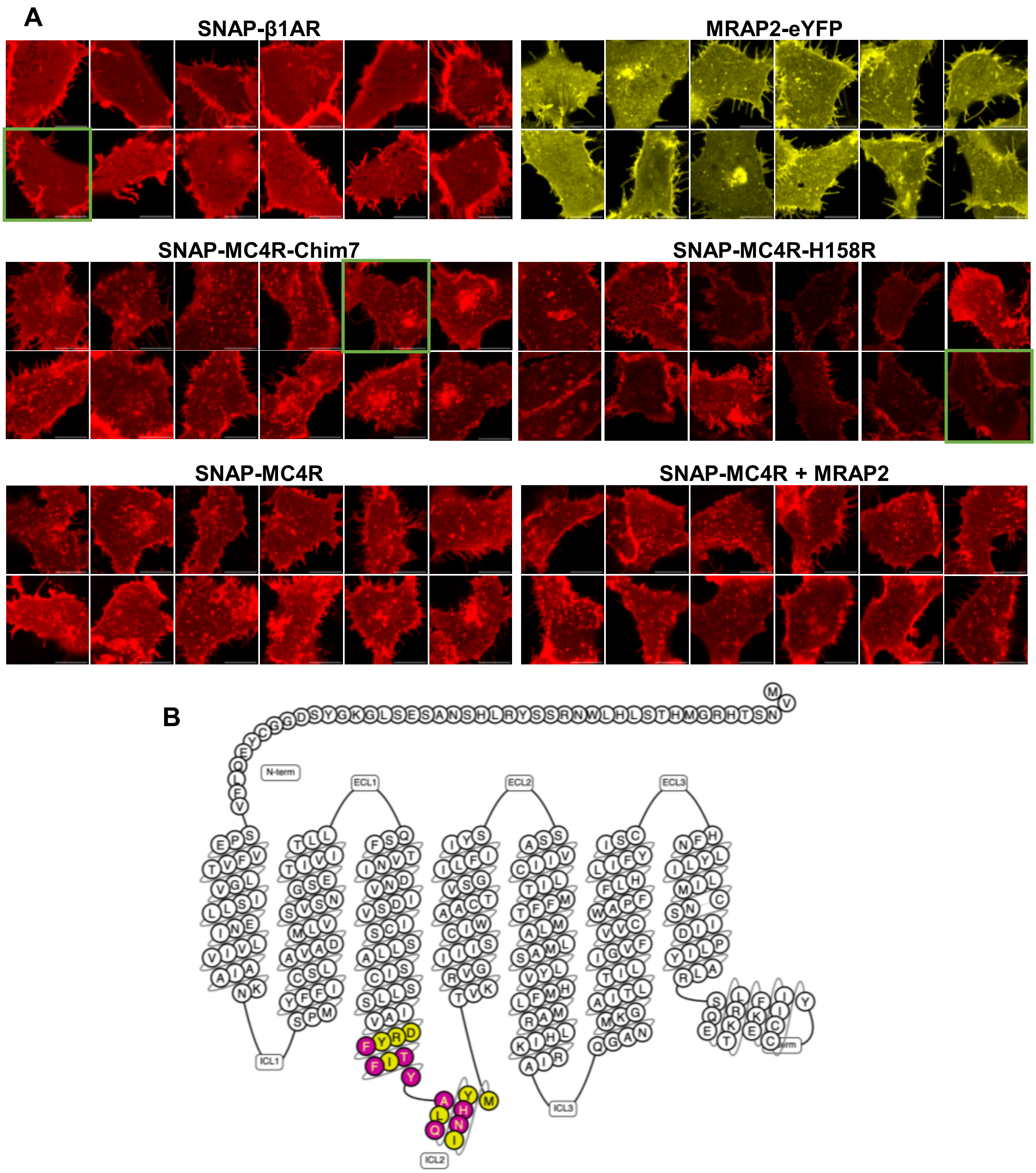

Supplementary Figure 8: Control datasets for molecular brightness experiments. (A) Representative confocal micrographs of HEK293 cells expressing the constructs used throughout the manuscript for fluorescence microscopy, all labeled with SNAP-surface Alexa 647. Green borders indicate the pictures that were used for **Figure 7. (B)** Snake plot diagram illustrating the mutated residues in ICL2 loops to generate MC4Rchim.

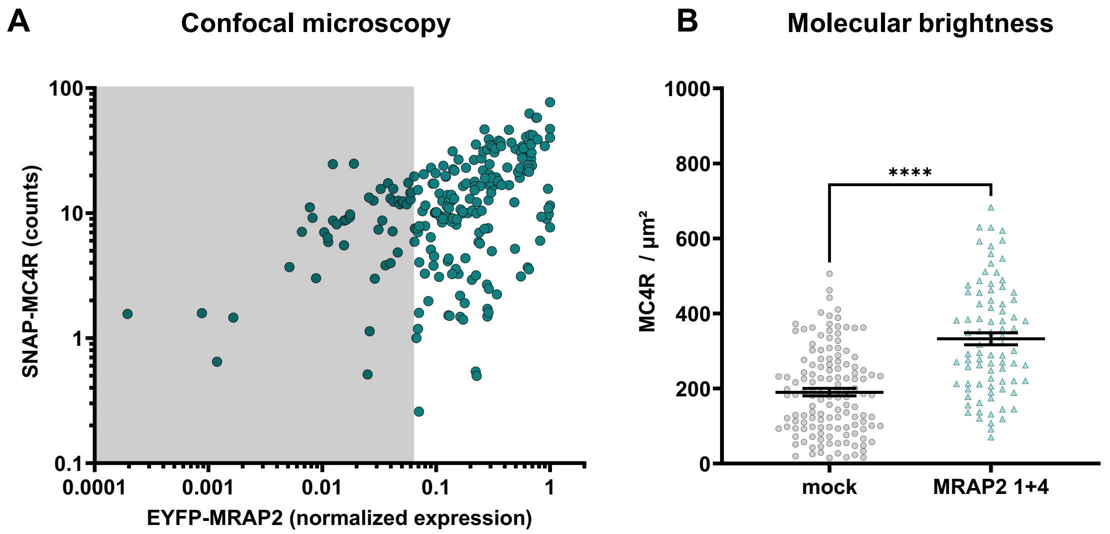

Supplementary Figure 9: Effect of MRAP2 expression on MC4R membrane expression was assessed in two independent ways. (A) Labelling of SNAP-MC4R using a membrane impermeable dye (SNAP-surface549) allows correlating MC4R membrane abundance to overall expression of eYFP-MRAP2 (normalized), based on intensity counts from confocal micrographs of HEK293 cells transfected with SNAP-MC4R in the presence or absence of eYFP-MRAP2 at a 1+4 ratio, using only the cells that received 0.5 nM AF647-NDP-α-MSH. Threshold for non-transfected cells (in the dark section) was chosen by visual inspection of the cells. **(B)** A closer quantification of receptor expression in cells with and without MRAP2 (1+4) was conducted using molecular brightness, which allows to count the number of emitters per unit area of the plasma membrane. Measurements conducted using molecular brightness. Mean and ± SEM are displayed on top of individual points reflecting receptor numbers from mean of ROIs measured within individual cells. SNAP-MC4R (135 cells, 6 transfections) and SNAP-MC4R + MRAP2 (n=4 transfections, 85 cells). Statistical analysis was performed using an unpaired two tailed t-test (****: *p*-value < 0.0001).

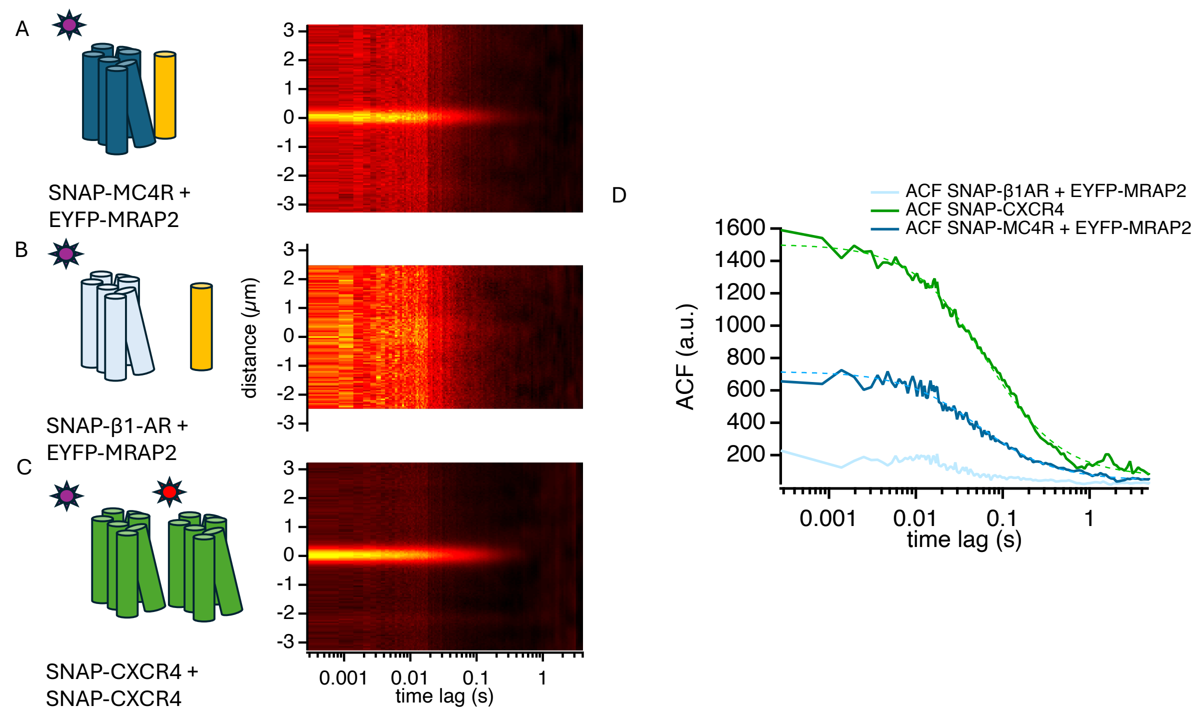

Supplementary Figure 10: Molecular interaction between MC4R and MRAP2 probed using Spatial-temporal Fluorescence Cross-Correlation Spectroscopy (ccSTICS): (A) Average ccSTICS function of the interaction between SNAP-MC4R, labeled with Snap-surface Alexa647, and eYFP-MRAP2, displaying the presence of co-diffusion (n=3 independent transfections, 15 cells). **(B)** Average ccSTICS function of the interaction between SNAP-β1AR, labeled with Snap-surface Alexa647, and eYFP-MRAP2, displaying the absence of any co-diffusion. (n=2 independent transfections, 13 cells). **(C)** Average ccSTICS function of the interaction between SNAP-CXCR4 protomers, stoichiometrically labeled with Snap-surface Alexa549 and Alexa647, displaying the presence of co-diffusion consistent with the dimeric nature of this receptor. (n=2 independent transfections, 19 cells). **(D)** Resulting cross-correlation functions, illustrating comparable diffusion times for the CXCR4 homodimer (D=0.34 $\mu m^2/s$) and the MC4R-MRAP2 heterodimer (0.43 $\mu m^2/s$).

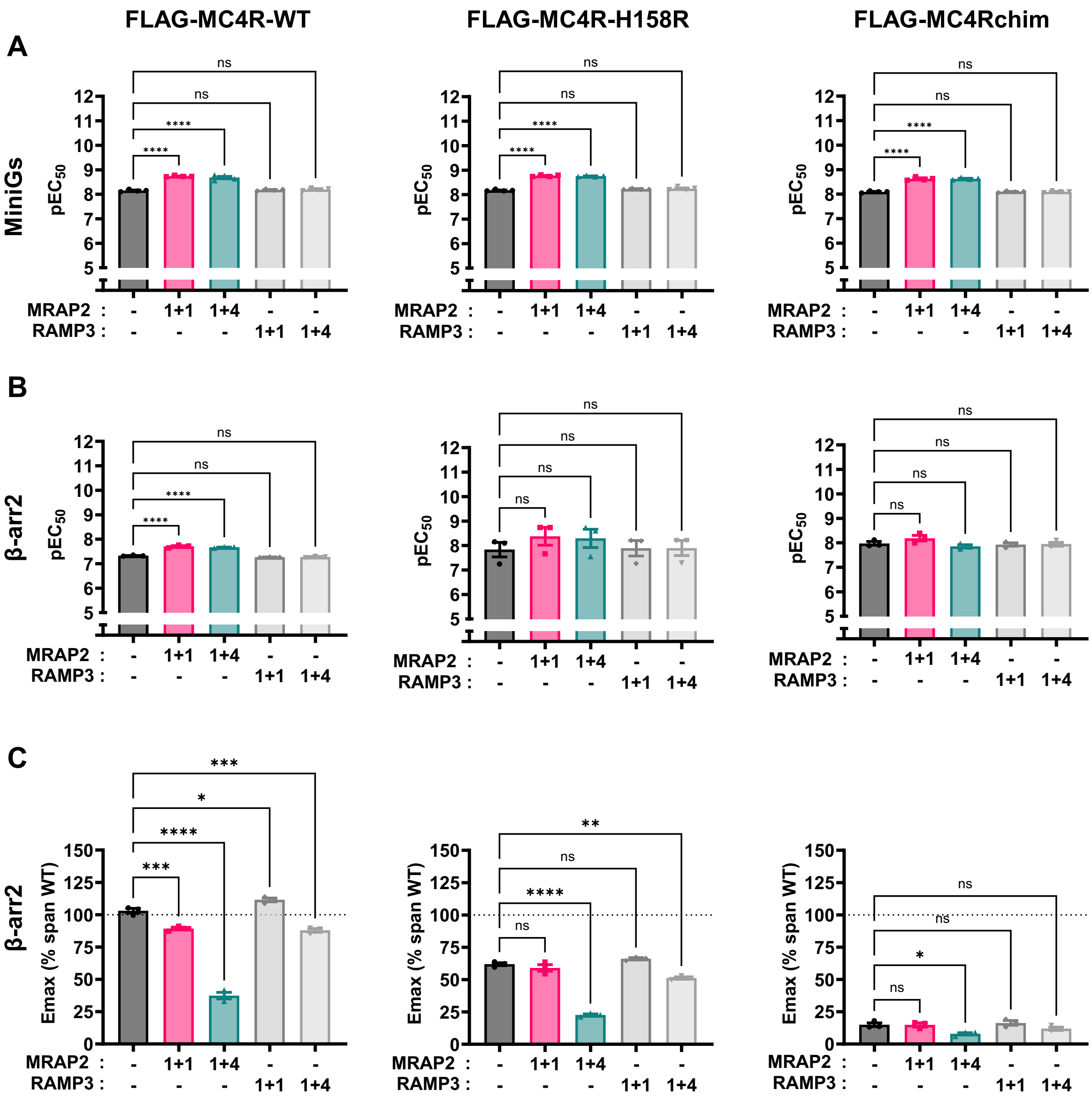

Supplementary Figure 11: Parameters for concentration-response curves of the MC4R-WT, MC4R-H158R and MC4Rchim in Figure 8. (A) pEC_{50} values and **(B)** E_{max} values from the Rluc8-miniGs recruitment BRET experiments. Results are represented as mean ± SEM, in which individual data point from the four individual experiments are shown. **(C)** E_{max} values from the β-arrestin2 recruitment BRET experiments. Results are represented as mean ± SEM, in which individual pEC_{50} or E_{max} data point from the three individual experiments are shown. Statistical analysis was performed using ordinary one-way ANOVA with Dunnett's multiple comparisons post-hoc test (* = $p < 0.05$ in which adjusted *p*-value for WT mock vs RAMP3 1+1 E_{max} is 0.0188 and adjusted *p*-value for Chim mock vs MRAP2 1+4 E_{max} is 0.0190 ; ** = $p = 0.0014$, *** = $p < 0.001$ in which adjusted *p*-value for WT mock vs MRAP2 1+1 E_{max} is 0.0007 and adjusted *p*-value for WT mock vs RAMP3 1+4 E_{max} is 0.0003; **** = $p < 0.0001$).

```
hMRAP1   . . . . . . . M A N G T N A S A P Y Y S Y E Y Y L D Y L D L I P V D E K K L K A    33
hMRAP2   M S A Q R L I S N R T S Q Q S A S N S D Y T W E Y E Y Y E I G P V S F E G L K A    40

                      Transmembrane helix
hMRAP1   H K H S I V I A F W V S L A A F V V L L F L I L L Y M S W S A S P Q M R N S P K    73
hMRAP2   H K Y S I V I G F W V G L A V F V I F M F F V L T L L T K T G A P H Q D N A E S    80

hMRAP1   H H Q T C P W S H G L N L H L C I Q K C L P C H R E P L A T S Q A Q A S S V E P    113
hMRAP2   S E K R F R M N S F V S D F G R P L E P D K V F S R Q G N E E S R S L F H C Y I    120

hMRAP1   G S R T G P D Q P L R Q E S S S T L P L G G F Q T H P T L L W E L T L N G G P L    153
hMRAP2   N E V E R L D R A K A C H Q T T A L D S D V Q L Q E A I R S S G Q P E E E L N R    160

hMRAP1   V R S K P S E P P P G D R T S Q L Q S . . . . . . . . . . . . . . . . . . . . .    172
hMRAP2   L M K F D I P N F V N T D Q N Y F G E D D L L I S E P P I V L E T K P L S Q T S    200

hMRAP1   . . . . .                                                                          172
hMRAP2   H K D L D                                                                          205
```

Supplementary Figure 12: Alignment of the human MRAP1 and MRAP2 sequences. This alignment was performed manually using MRAP1 isoform α (NCBI RefSeq: NP_001366157.1) and MRAP2 isoform 1 (NCBI RefSeq: NP_001333471.1). Color coding reflects physicochemical properties of the amino acid side chains: green - hydrophobic non-polar (A, G, I, L, M, V); turquoise - hydrophobic aromatic (F, W, Y); blue – basic (H, K, R); purple - amidic; magenta – hydroxylic (Q, N); red – acidic (D, E); orange - cysteine; grey - proline. Residues with similar chemical function are highlighted (sequence similarity). Of note, this (structural) alignment without gap is different to recently proposed [15], where the MRAP1 ‘$_{18}$LDYL$_{21}$’ motif in the N-terminus is suggested to be an MRAP1 specific insertion (or is deleted in MRAP2) compared to the MRAP2 sequence.

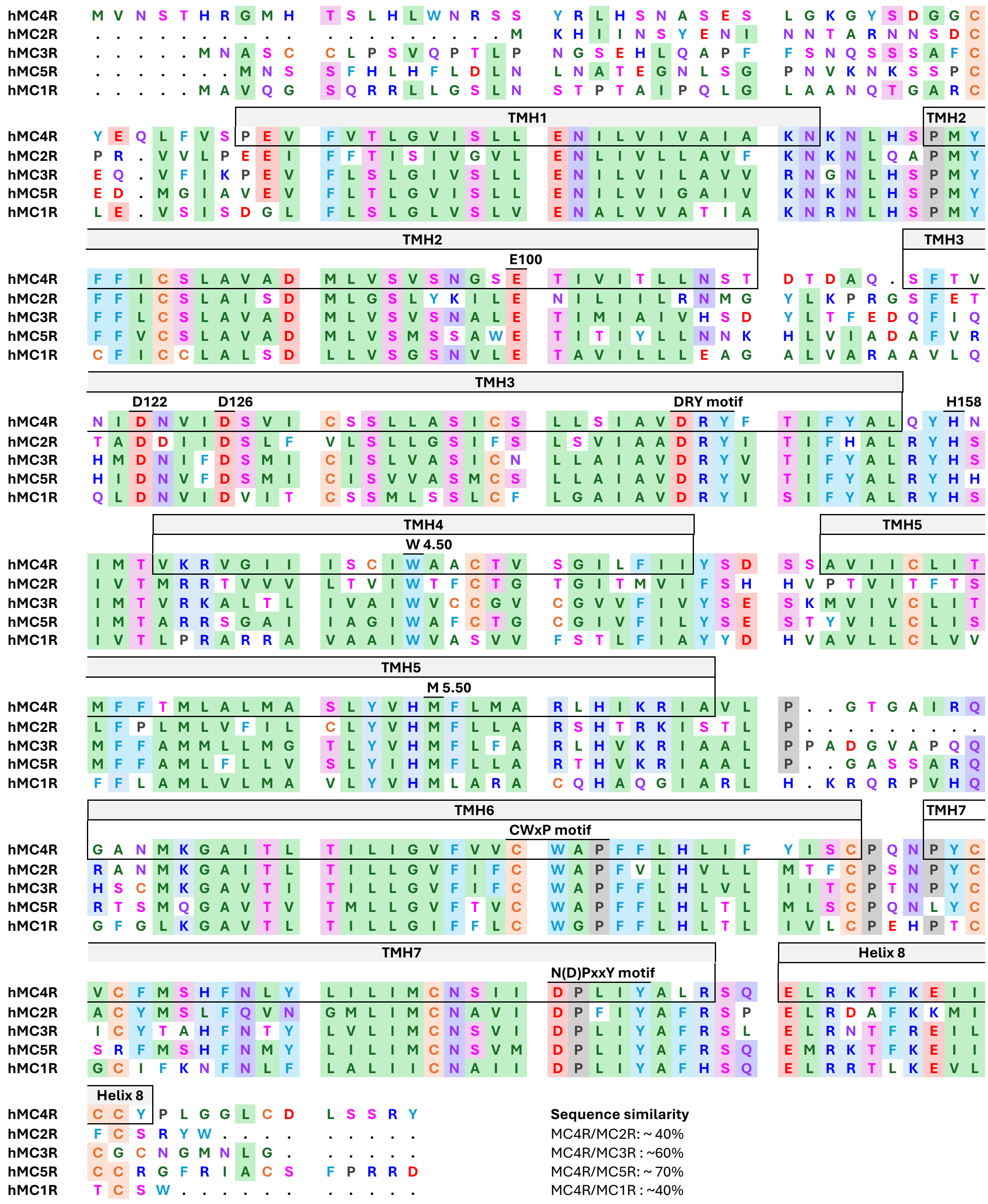

Supplementary Figure 13: Alignment of the human melanocortin receptors sequences. This alignment was performed manually using MC4R (NCB RefSeq: NP_005903.2), MC2R/ACTHR (NCBI RefSeq: NP_000520.1), MC3R (NCB RefSeq: NP_063941.3), MC5R (NCBI RefSeq: NP_005904.1) and MC1R/MSHR (NCBI RefSeq: NP_002377.4). Color coding reflects physicochemical properties of the amino acid side chains: green - non-polar (A, G, I, L, M, V); turquoise - hydrophobic aromatic (F, W, Y); blue – basic (H, K, R); purple - amidic; magenta - hydroxylic (Q, N); red - acidic (D, E); orange - cysteine; grey - proline. Residues are highlighted when at least three of them are similar in chemical function (sequence similarity). Additionally, structural lengths of MC4R transmembrane helices (TMH), conserved structural motifs (DRY, CWxP, N(D)PxxY), the negatively charged amino acids involved in calcium binding (E100, D122 and D126), and the position of the histidine mutated in the MC4R-H158R mutant are indicated.

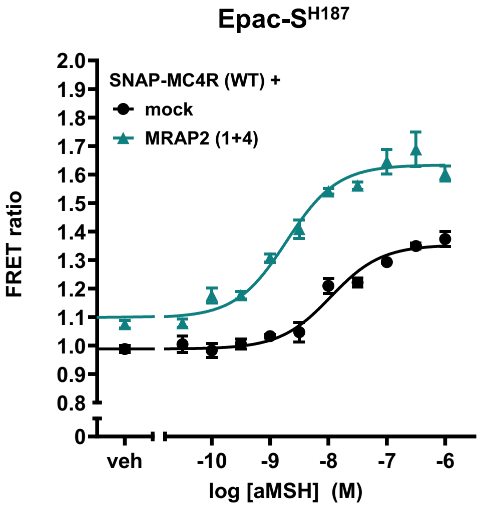

Supplementary Figure 14: Concentration response curve of FRET ratio (donor/FRET), as a function of α-MSH concentration for N7 cells transfected with MC4R, EPAC-SH187 and co-transfected with either MRAP2 (1+4) or a mock plasmid. Data are not normalised and shown as mean and standard error of the mean (±SEM) to emphasize the change in constitutive activity between the two conditions.
