# Supplementary material for: MRAP2 modifies the signaling and oligomerization state of the melanocortin-4 receptor
Source: Nat Commun. 2025 Sep 25;16:8324. doi: 10.1038/s41467-025-63988-w (PMC12462500; doi:10.1038/s41467-025-63988-w)
Supplement: Supplementary file 2 — Reporting Summary [file 41467_2025_63988_MOESM2_ESM.pdf]

nature portfolio

Corresponding author(s): MB, PMC, PA, HB

Last updated by author(s): Aug 28, 2025

Reporting Summary

Nature Portfolio wishes to improve the reproducibility of the work that we publish. This form provides structure for consistency and transparency in reporting. For further information on Nature Portfolio policies, see our Editorial Policies and the Editorial Policy Checklist.

Statistics

For all statistical analyses, confirm that the following items are present in the figure legend, table legend, main text, or Methods section.

n/a	Confirmed	
☐	☒	The exact sample size (*n*) for each experimental group/condition, given as a discrete number and unit of measurement
☐	☒	A statement on whether measurements were taken from distinct samples or whether the same sample was measured repeatedly
☐	☒	The statistical test(s) used AND whether they are one- or two-sided<br>*Only common tests should be described solely by name; describe more complex techniques in the Methods section.*
☐	☒	A description of all covariates tested
☐	☒	A description of any assumptions or corrections, such as tests of normality and adjustment for multiple comparisons
☐	☒	A full description of the statistical parameters including central tendency (e.g. means) or other basic estimates (e.g. regression coefficient) AND variation (e.g. standard deviation) or associated estimates of uncertainty (e.g. confidence intervals)
☐	☒	For null hypothesis testing, the test statistic (e.g. *F*, *t*, *r*) with confidence intervals, effect sizes, degrees of freedom and *P* value noted<br>*Give P values as exact values whenever suitable.*
☒	☐	For Bayesian analysis, information on the choice of priors and Markov chain Monte Carlo settings
☒	☐	For hierarchical and complex designs, identification of the appropriate level for tests and full reporting of outcomes
☒	☐	Estimates of effect sizes (e.g. Cohen's *d*, Pearson's *r*), indicating how they were calculated

Our web collection on statistics for biologists contains articles on many of the points above.

Software and code

Policy information about availability of computer code

Data collection	Leica LAS X (v3.5.5) for confocal and STICS imaging using the Leica SP8 microscope.<br><br>Zeiss Zen Blue software for confoal acquisition using the Zeiss LSM800 microscope<br><br>NIS-Elements (v5.21) for TIRF microscopy on the Nikon Eclipse Ti2.<br><br>Synergy Neo2 software (BioTek) for FRET and HTRF/IP-One assay readouts.<br><br>CLARIOstar Plus control software (BMG Labtech) for NanoBiT assays.<br><br>MicroBeta2 Workstation software (PerkinElmer) for scintillation proximity assay (SPA) measurements.<br><br>Tecan Spark Control Software (v2.3) for sCFP3A-EPAC-Venus FRET experiments.<br><br>FLEX Station II software (Molecular Devices) for fluorescence acquisition in BRET-based assays.<br><br>ImageJ/Fiji (v1.53 and later) was used to manually define ROIs and extract intensity data from microscopy and time-lapse imaging files.
Data analysis	GraphPad Prism (v10.3.1, GraphPad Software, San Diego, CA, USA) was used for all statistical analyses, including curve fitting (e.g., EC_{50} and E_{max}), ANOVA, and normalization calculations for dose-response and binding assays<br>.

IgorPro (Wavemetrics) Version: 9.05 (Build 56551)

A python version of the code used to generate the STICS functions can be found on github https://github.com/LynnLangstrumpf/Master-Thesis
Microsoft Excel 365 Version 16.95.1 (25031528)

ImageJ/Fiji (v1.53 and later) was used for fluorescence quantification, ROI analysis, background correction, and image processing including time-lapse, z-stack projections, and molecular brightness (SpIDA) assessments.

LAS X (v3.5.5, Leica Microsystems) was used for initial data inspection and preprocessing of confocal imaging files.

NIS-Elements (v5.21, Nikon) for analysis of TIRF microscopy data, including time-series fluorescence quantification.

MicroBeta2 Workstation software (PerkinElmer) and CLARIOstar Plus analysis suite (BMG Labtech) were used for radioligand binding SPA and NanoBiT assay data quantification respectively.

Tecan Spark Control Software (v2.3) was used for emission ratio calculations in FRET-based assays.

MATLAB R2023a (MathWorks) and custom Python 3.9 scripts were employed for Spatial-Temporal Image Correlation Spectroscopy (STICS) analysis, adapted from published methods (Bathe-Peters et al. 2020), including detrending, 2D autocorrelation fitting, and diffusion coefficient extraction.

SYBYL-X 2.0 (Certara, NJ, US)

For manuscripts utilizing custom algorithms or software that are central to the research but not yet described in published literature, software must be made available to editors and reviewers. We strongly encourage code deposition in a community repository (e.g. GitHub). See the Nature Portfolio guidelines for submitting code & software for further information.

Data

Policy information about availability of data

All manuscripts must include a data availability statement. This statement should provide the following information, where applicable:

- Accession codes, unique identifiers, or web links for publicly available datasets
- A description of any restrictions on data availability
- For clinical datasets or third party data, please ensure that the statement adheres to our policy

The source data for the images, charts and graphs of Figures 1-8 are provided as Source Data files. The raw microscopy data underpinning the charts and graphs reported in this manuscript are available under restricted access due to their very large size, and can be obtained by request to the corresponding author. Please allow for two weeks to address the request, and data will be shared via an appropriate online repository for the duration of a week. Source Data are provided with this paper.

Research involving human participants, their data, or biological material

Policy information about studies with human participants or human data. See also policy information about sex, gender (identity/presentation), and sexual orientation and race, ethnicity and racism.

Reporting on sex and gender	*Use the terms sex (biological attribute) and gender (shaped by social and cultural circumstances) carefully in order to avoid confusing both terms. Indicate if findings apply to only one sex or gender; describe whether sex and gender were considered in study design; whether sex and/or gender was determined based on self-reporting or assigned and methods used. Provide in the source data disaggregated sex and gender data, where this information has been collected, and if consent has been obtained for sharing of individual-level data; provide overall numbers in this Reporting Summary. Please state if this information has not been collected. Report sex- and gender-based analyses where performed, justify reasons for lack of sex- and gender-based analysis.*
Reporting on race, ethnicity, or other socially relevant groupings	*Please specify the socially constructed or socially relevant categorization variable(s) used in your manuscript and explain why they were used. Please note that such variables should not be used as proxies for other socially constructed/relevant variables (for example, race or ethnicity should not be used as a proxy for socioeconomic status). Provide clear definitions of the relevant terms used, how they were provided (by the participants/respondents, the researchers, or third parties), and the method(s) used to classify people into the different categories (e.g. self-report, census or administrative data, social media data, etc.) Please provide details about how you controlled for confounding variables in your analyses.*
Population characteristics	*Describe the covariate-relevant population characteristics of the human research participants (e.g. age, genotypic information, past and current diagnosis and treatment categories). If you filled out the behavioural & social sciences study design questions and have nothing to add here, write "See above."*
Recruitment	*Describe how participants were recruited. Outline any potential self-selection bias or other biases that may be present and how these are likely to impact results.*
Ethics oversight	*Identify the organization(s) that approved the study protocol.*

Note that full information on the approval of the study protocol must also be provided in the manuscript.

Field-specific reporting

Please select the one below that is the best fit for your research. If you are not sure, read the appropriate sections before making your selection.

☒ Life sciences ☐ Behavioural & social sciences ☐ Ecological, evolutionary & environmental sciences

For a reference copy of the document with all sections, see nature.com/documents/nr-reporting-summary-flat.pdf

Life sciences study design

All studies must disclose on these points even when the disclosure is negative.

Sample size	No statistical method was used to predetermine sample size.
Data exclusions	Radioligand binding experiments were repeated independently five times, but two datasets were excluded as the negative controls were not correct.
Replication	All experiments reported in the main Figures were repeated independently in at least three biological replicates with similar results.
Randomization	The experiments were not randomized
Blinding	. The investigators were not blinded to allocation during experiments and outcome assessment.

Reporting for specific materials, systems and methods

We require information from authors about some types of materials, experimental systems and methods used in many studies. Here, indicate whether each material, system or method listed is relevant to your study. If you are not sure if a list item applies to your research, read the appropriate section before selecting a response.

Materials & experimental systems

n/a	Involved in the study	
☐	☒	Antibodies
☐	☒	Eukaryotic cell lines
☒	☐	Palaeontology and archaeology
☒	☐	Animals and other organisms
☒	☐	Clinical data
☒	☐	Dual use research of concern
☒	☐	Plants

Methods

n/a	Involved in the study	
☒	☐	ChIP-seq
☒	☐	Flow cytometry
☒	☐	MRI-based neuroimaging

Antibodies

Antibodies used	anti-IP1-Cryptate antibody
Validation	Albeit this specific antibody was spelled out, it is part of a Revvity (Ex Cisbio) kit that has been used by dozens of labs, and is now reported in 141 publications. On Revvity's website, this is cited that this kit has been used in 141 publications. https://www.revvity.com/de-en/product/htrf-ip-one-gq-kit-1k-pts-62ipapeb#product-overview

Eukaryotic cell lines

Policy information about cell lines and Sex and Gender in Research

Cell line source(s)	HEK293T: ECACC 96121229, Sigma-Aldrich<br><br>HEK293Adherent (HEK293AD): R70507, Thermo Fisher<br><br>HEK293-SL: Derived from Ad5-transformed HEK293 cells, provided by S. Laporte (McGill University)<br>Hek293 sex is female.<br><br>β-arrestin1 and β-arrestin2 double knock-out cells: CRISPR-modified from HEK293-SL cells<br><br>mHypoE-N7: Cedarlane Labs (CVCL_D462)<br>N7 sex is male.
Authentication	No specific authentication procedures were reported for the cell lines used in this study.

Authentication	
Mycoplasma contamination	All HEK293 variants were routinely tested for mycoplasma using the MycoAlert Mycoplasma Detection Kit (Lonza) or PCR-based Mycoplasma Detection Kit (ABM #G238), and confirmed negative.
Commonly misidentified lines (See ICLAC register)	None of the cell lines used in the study are known to be commonly misidentified.

Plants

Seed stocks	*Report on the source of all seed stocks or other plant material used. If applicable, state the seed stock centre and catalogue number. If plant specimens were collected from the field, describe the collection location, date and sampling procedures.*
Novel plant genotypes	*Describe the methods by which all novel plant genotypes were produced. This includes those generated by transgenic approaches, gene editing, chemical/radiation-based mutagenesis and hybridization. For transgenic lines, describe the transformation method, the number of independent lines analyzed and the generation upon which experiments were performed. For gene-edited lines, describe the editor used, the endogenous sequence targeted for editing, the targeting guide RNA sequence (if applicable) and how the editor was applied.*
Authentication	*Describe any authentication procedures for each seed stock used or novel genotype generated. Describe any experiments used to assess the effect of a mutation and, where applicable, how potential secondary effects (e.g. second site T-DNA insertions, mosiacism, off-target gene editing) were examined.*
